# Early and High SARS-CoV-2 Neutralizing Antibodies Are Associated with Severity in COVID-19 Patients from India

**DOI:** 10.4269/ajtmh.21-0014

**Published:** 2021-06-17

**Authors:** Shubham Shrivastava, Sonali Palkar, Jignesh Shah, Prajakta Rane, Sanjay Lalwani, Akhilesh Chandra Mishra, Vidya A. Arankalle

**Affiliations:** 1 Department of Communicable Diseases, Interactive Research School for Health Affairs, Bharati Vidyapeeth Deemed to be University, Katraj-Dhankawadi, Pune, India;; 2 Department of Community Medicine, Bharati Vidyapeeth Deemed to be University Medical College, Katraj-Dhankawadi, Pune, India;; 3 Department of Critical Care Medicine, Bharati Vidyapeeth Deemed to be University Medical College, Katraj-Dhankawadi, Pune, India;; 4 Department of Pediatrics, Bharati Vidyapeeth Deemed to be University Medical College, Katraj-Dhankawadi, Pune, India

## Abstract

Patients with SARS-CoV-2 infection have a wide spectrum of clinical presentations, from asymptomatic infection, to mild illness, to severe disease with recovery or fatal outcome. Immune correlates of protection are not yet clear. To understand the association between presence and titers of neutralizing antibodies (NAb) with recovery, we screened 82 COVID-19 patients classified in mild (*n* = 56) and severe (*n* = 26) disease groups on different days post onset of disease and 27 viral RNA–positive asymptomatic contacts examined within 1 week of the identification of index cases. Of 26 patients with severe disease, six died and 20 recovered. Anti-SARS-CoV-2 NAb levels in plasma and serum were measured using a plaque reduction neutralization test with live virus. The proportion of asymptomatic and symptomatic infections was 1:7.8 in males and 1:1 in females, with males predominating the severe disease group (21/26, 80.7%). At the time of presentation, NAb positivity and titers were comparable among groups with asymptomatic and mild infections. Notably, patients with severe disease exhibited higher NAb seropositivity and titers (25 of 26, 96.2%; 866 ± 188) than those in the mild category (39 of 56, 69.6%; 199 ± 50, *P* < 0.0001) and asymptomatic individuals (21 of 27, 77.8%; 124 ± 28, *P* = 0.0002). Within first 2 weeks of onset, NAb titers were significantly higher among patients with severe disease than those with mild presentation. Our data suggest that irrespective of fatal outcome, progression to disease severity was associated with induction of early and high levels of NAb. In our patient series, clinical disease, severity and fatality were predominantly seen in males. The role of NAbs in immunopathogenesis or protection needs to be defined.

## INTRODUCTION

The continued rapid spread of severe acute respiratory syndrome coronavirus 2 (SARS-CoV-2) causing coronavirus disease 2019 (COVID-19) has affected more than 84 million people worldwide.^1^ Approximately 80% of SARS-CoV-2 infections are asymptomatic or mild, 15% are severe requiring oxygen, whereas 5% are critical, requiring ventilation.^2^ The overall case fatality rate is between 3% and 4%. Disease severity and mortality is shown to be higher in the elderly and individuals with comorbidities.^3^^,^^4^

With the unprecedented spread and magnitude of COVID-19, several vaccines have been rapidly developed, and some have been approved for immunization. To understand whether the immune response to different vaccines is protective, it is essential to understand immunologic response to natural infection and identify markers of protection. This would involve both humoral and cell-mediated immunity. Being a novel pathogen, methods need to be developed, validated, and used. For obvious reasons, antibody response has been the first global target. Several serological assays using recombinant viral proteins or inactivated whole virus were developed and used to characterize antibody responses induced by SARS-CoV-2 infection.^5^^–^^15^ On the basis of ELISA for immunoglobulin G (IgG) anti-SARS-CoV-2 detection, it was suggested that antibody response in COVID-19 patients is delayed.^16^^–^^18^ The present study was planned to estimate neutralizing antibody titers in patients with different clinical presentations.

## MATERIALS AND METHODS

### Human ethics approval.

This study was approved by Institutional Ethics Committee of Bharati Hospital and Research Center at Bharati Vidyapeeth Deemed University (IEC/2020/25). Informed written consent was obtained from each subject before participating in this study. This study was conducted in accordance with the ethical standards of the Helsinki Declaration of 1975, as revised in 2013.

### Study subjects and blood samples.

Patients seeking diagnosis and treatment at a special COVID center at Bharati Vidyapeeth (deemed to be university) hospital, a tertiary care hospital at Pune, India, were included in the study (April–June 2020). Diagnosis of COVID-19 was carried out by the hospital as per the existing guidelines of the government of India. The study included 82 viral RNA–positive, confirmed COVID-19 patients of mild (*n* = 56) and severe (*n* = 26) disease categories and 27 asymptomatic contacts of COVID-19 patients identified as part of the government’s aggressive contact tracing program. Patients who did not require oxygen supplementation were classified in the mild disease group. Patients requiring admission in intensive care units with oxygen saturation levels below 93% at room air were categorized in the severe disease group. Initial blood samples from all symptomatic patients were collected at the time of admission. Follow-up blood samples from patients with mild disease (*n* = 6) were collected 6 to 7 days after admission. For one mild disease patient, a follow-up sample was collected 31 days after admission. For severe disease patients (*n* = 18), blood samples were collected either on alternate days or at the time of discharge that varied from 1 to 3 weeks. Blood samples were collected from viral RNA–positive asymptomatic contacts of COVID-19 index cases, within 1 week of RNA positivity in the asymptomatic contacts and the index case. Serum and plasma samples were stored at –80°C in aliquots until used. Additionally, plasma samples collected from 61 blood donors before the emergence of SARS-CoV-2 (2017–2019) and stored similarly were used to determine cutoff value for a positive plaque reduction neutralization test (PRNT) test.

### Cell culture.

Vero CCL81 cells were procured from ATCC (Manassas, VA). Vero cells were cultured in minimum essential medium (MEM; Gibco, Waltham, MA) supplemented with 10% fetal bovine serum (FBS, Gibco) and 100 units/mL of penicillin-streptomycin and kept at 37°C in 5% CO_2_ incubator.

### Virus.

SARS-CoV-2 virus (8004/IND/2020/IRSHA PUNE, accession number MT416726) was isolated from nasopharyngeal swab of a COVID-19 patient positive for viral RNA by RdRp based real-time polymerase chain reaction (PCR). Propagation and large-scale production of SARS-CoV-2 was performed in biosafety level (BSL)-3 facility according to the India government’s Department of Biotechnology guidelines. Vero cells were seeded in T-175 cm^2^ flask at a density of 1 × 10^6^ cells/mL. The next day, cells were infected with SARS-CoV-2 virus at a multiplicity of infection of 0.001. After 1 hour of adsorption at 37°C in humidified incubator with 5% CO_2_, inoculum was removed, cells were washed twice to remove input virus, and 20 mL of fresh MEM containing 2% FBS and antibiotics was added to each T-175 cm^2^ flask. Flasks were observed daily for cytopathic effect (CPE), and virus was harvested when 80% to 90% of cells were showing CPE. Culture supernatants were collected and centrifuged at 2,000 rpm for 10 minutes at 4°C to remove the cell debris; supernatants were aliquoted and stored at –80°C until used.

### Plaque reduction neutralization test.

For PRNT, 1 day before infection, 1 × 10^5^ cells/mL were seeded in a 24-well plate using MEM containing 10% FBS and antibiotics. Serum samples diluted at a ratio of 1:5 were heat inactivated for 30 minutes at 56°C. Four-fold serial dilution was performed and mixed with equal volume of 40 to 80 pfu of virus. The serum–virus mixture was incubated for 1 hr at 37°C in humidified incubator with 5% CO_2_. After incubation, 100 μL of the mixture was added in duplicate to 24-well plate and incubated for 1 hour at 37°C in humidified incubator with 5% CO_2_. After incubation, 1 mL of 1% overlay media containing MEM, Aquacide-II (Merck, Darmstadt, Germany), 2% FBS and antibiotics was added to Vero cell monolayer. Plates were incubated for 5 days at 37°C in humidified incubator with 5% CO_2_. At 5 days postinfection, overlay medium was discarded, cells were fixed using 3.7% formaldehyde, and after washing with phosphate-buffered saline, cells were stained using 1% crystal violet. Plates were washed and air-dried. Plaques were counted manually, and PRNT_50_ titer was calculated using Karber’s formula, log10 PRNT_50_ = m − Δ (Σp − 0.5), where m is the log10 of the highest dilution and Δ is the constant interval between dilutions expressed as log10, as previously described.^19^

### Statistical analysis.

Data analysis was performed using GraphPad Prism version 5.0. Mann-Whitney test was performed to compare PRNT_50_ titers among different groups (e.g., disease severity, gender, post onset of disease [POD], and POD in different disease groups). Wilcoxon signed rank paired test was performed to compare PRNT_50_ titers at the time of admission and follow-up in mild COVID-19 patients. A *P* value < 0.05 was considered statistically significant.

## RESULTS

The patients’ characteristics are summarized in Table 1. Of the 109 patients studied, 27 were asymptomatic (age range: 16–66, median 40), 56 had mild disease (age range: 18–79, median 42), and 26 patients (age range: 28–70, median 42) suffered from severe disease and required hospitalization in high-dependency or intensive care units. Of these, four needed O_2_ administration, and 16 required mechanical ventilator support. Comorbidities (cardiovascular disease, diabetes, hypertension, and chronic lung disease) were reported by 15 of 26 patients (57.7%) with severe disease including four of six patients (66.7%) with fatal outcome. The proportion of comorbidities was significantly lower in mild disease patients (11 of 56, 19.6%; *P* = 0.001) and asymptomatic individuals (5 of 27, 18.5%; *P* = 0.008). In our patient series, a significant proportion of males were symptomatic (62 of 82, 75.6%; *P* < 0.001) and presented with severe disease (21 of 26, 80.8%; *P* < 0.001), whereas females predominated asymptomatic infection (19 of 27, 70.4%; *P* = 0.0065). The proportion of asymptomatic and symptomatic infections was 1:7.8 in males and 1:1 in females. Five males (aged 30, 42, 55, 60, and 65) and one female (aged 59) succumbed to the infection.

**Table 1:** Patient’s characteristics and symptoms at presentation

Study groups		Asymptomatic (*n* = 27)	Symptomatic (*n* = 82)*
Age	Age in years (median)	16–66 (40)	18–79 (42)
Gender, n (%)			
	Male	8 (29.6)	62 (75.6)
	Female	19 (70.4)	20 (24.4)
			
Disease severity, n (%)	Mild	NA	56 (68.3)
	Severe		26 (31.7)
			
Symptoms, n (%)	Fever	NA	47 (57.3)
	Cough		39 (47.6)
	Dyspnea		34 (41.5)
	Sore throat		24 (29.3)
	Mechanical ventilation		16 (19.5)
			
Comorbidity, n (%)	Yes	5 (18.5)	28 (34.1)
	No	22 (81.5)	54 (65.9)
	Cardiovascular disease	5 (18.5)	14 (17.1)
	Hypertension	2 (7.4)	8 (9.7)
	Diabetes	1 (3.7)	14 (17.1)

NA = not applicable.

* Symptomatic infection in males and females (75.6% vs. 24.4% *P* < 0.001); gender predisposition in asymptomatic vs. symptomatic infection (29.6% vs. 75.6% in males, *P* < 0.001; 70.4% vs. 24.4% in females, *P* < 0.001).

To determine NAb titers, we first standardized PRNT_50_ using live SARS-CoV-2 (8004/IND/2020/IRSHA-Pune) isolated in our laboratory. For this, plasma from healthy donors collected in 2017 and a PCR-confirmed COVID-19 patient during convalescence (27 POD) were used as negative and positive controls, respectively.

### Neutralizing antibodies in healthy blood donors examined before COVID-19.

To assess specificity of the standardized PRNT assay, plasma and serum samples collected from 61 blood donors in 2017–2019 were tested for the presence of NAbs. All donors were found to be nonreactive at 1:10 dilution. We therefore decided to use titer of ≥ 10 as the criteria of antibody positivity in PRNT.

### Neutralizing antibody titers in COVID-19 patients.

**Figure 1.**
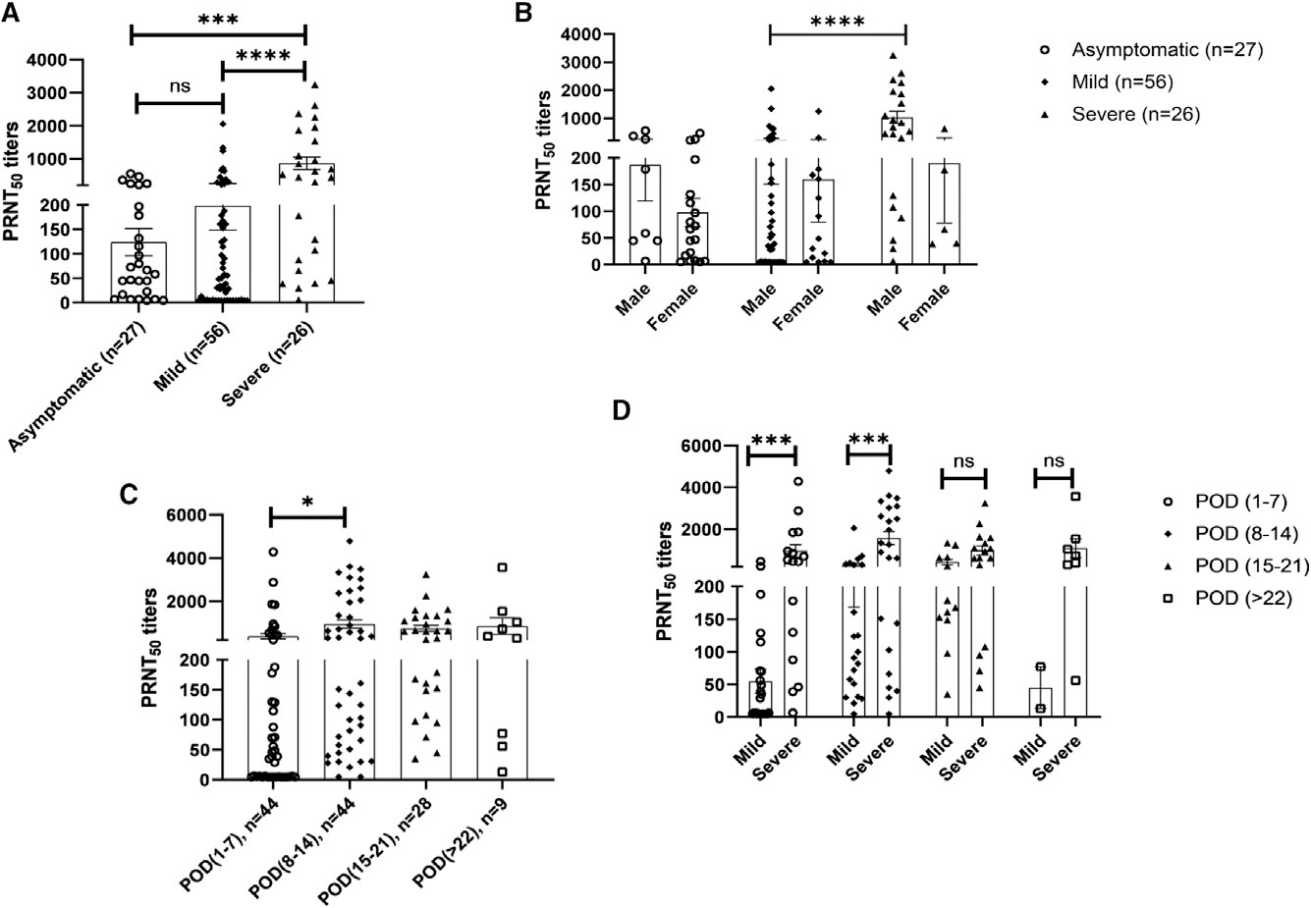
Neutralizing antibody titers in SARS-CoV-2 patients with (**A**) different clinical presentations, (**B**) in males versus females among different clinical presentations, (**C**) at different days post onset of disease (POD), and (**D**) comparison of neutralizing antibody titers between mild and severe patients at different POD. The data are presented as dot plots with bar representing the mean ± SEM in each group. Each dot represents a single sample. *P* values were calculated using Mann-Whitney test. *P < 0.05, **P < 0.01, ***P < 0.001, ****P < 0.0001.

At the time of first sampling, NAb seropositivity was comparable among asymptomatic individuals (21 of 27, 77.8%) and mild disease groups (39 of 56, 69.6%; *P* = 1.0). However, seropositivity in the severe disease patients (25 of 26, 96.15%) was higher than the mild disease and asymptomatic categories. Similar to seropositivity, NAb titers were significantly higher in severe disease patients (866 ± 188) compared with the mild (199 ± 50, *P* < 0.0001) and asymptomatic (124 ± 28, *P* = 0.0002) categories (Figure 1A). Comparable titers were observed in mild-disease patients and asymptomatic individuals (*P* = 0.32; Figure 1A). Gender-based comparison revealed that NAb titers in the mild disease were independent of patient gender (*P* = 0.79; Figure 1B). In the severe disease patients, although titers were higher in males (*n* = 21, 1027 ± 217), the difference was insignificant (*P* = 0.06), probably because of the small number of female patients (*n* = 5, 190 ± 112) in this category. Among males, the severe-disease group exhibited significantly higher NAb titers than those with mild disease (*P* < 0.0001). In contrast, titers among severe and mild disease categories were comparable in females (*P* = 0.39; Figure 1B).

Next, we examined the relationship of NAb titers with the duration between onset of clinical symptoms and sample collection, irrespective of disease category (Figure 1C). During the first week, 61.4% (27 of 44) COVID-19 patients were seropositive, increasing to 95.4% (42 of 44) during the second week. A significant increase in NAb titers was observed during this period (*P* = 0.02). Subsequently, 100% seropositivity with comparable NAb titers was seen. When disease severity and duration were considered (Figure 1D), NAb titers in the severe-disease patients were significantly higher than in the mild patients during the first (*P* = 0.0003) and second (*P* = 0.0004) weeks after disease onset. Comparable titers were recorded during the third and fourth weeks.

### Dynamics of antibody titers during follow-up of COVID-19 patients.

**Figure 2.**
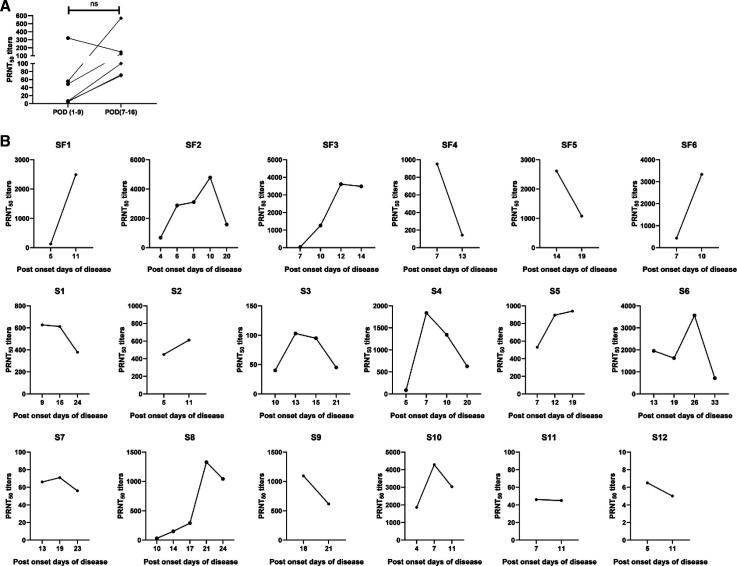
(**A**) Neutralizing antibody response in SARS-CoV-2 patients with mild disease at the time of admission (post onset of disease [POD] 1–9) and at follow-up (POD 7–16). The data are presented as line graphs with each line representing a single individual. *P* values were calculated using the Wilcoxon signed rank test. (**B**) Kinetics of neutralizing antibody response in individual COVID-19 patients with fatal outcome (SF1-SF6) and recovered (S1-S12).

Follow-up samples collected from seven mild-disease patients and 18 severe-disease patients were tested for neutralizing antibody titers (Figure 2). For six patients with mild disease, samples were collected at the time of admission and 6 to 7 days later. Three patients were seropositive at admission (POD 1–9), whereas the other three seroconverted at follow-up (POD 7–16) (Figure 2A). For one mild disease patient, NAb titers rose from 35 (POD = 7) to 77 (POD = 31). Figure 2B depicts modulation of NAb titers in 18 severe-disease patients, including six with fatal outcome. Although the response of individual patients varied, modulation pattern was independent of fatal outcome. The differential responses included 1) high NAb titers during the first week, increasing further during the second week (*n* = 5); 2) similar NAb titers at the time of admission and follow-up (*n* = 4); 3) continued high titers (*n* = 2); 4) gradual rise in one patient; 5) rapid decline in two patients; and 6) gradual decline in three patients. One patient remained seronegative with titers below 10 and viral RNA positive until day 11 (Figure 2B).

## DISCUSSION

Pathogenesis of severe COVID-19 with or without fatal outcome is not yet understood. Old age/existing comorbidities, decreased count of absolute T lymphocytes and raised interleukin-6 levels have been shown to be associated with severe viral infection.^2^^–^^4^ Our preliminary study reveals a clear association between neutralizing antibody titers and disease severity among Indian COVID-19 patients.

Antibody positivity against other human coronaviruses is not known for the Pune population, in which this study was undertaken. To determine cross reactivity and cutoff value for a positive PRNT, we screened stored serum samples from 61 blood donors collected before the emergence of the pandemic (2017–2019). At the minimum serum dilution of 1:10, all the blood donors were nonreactive, indicating absence or very low cross reactivity. A titer ≥ 10 was considered as the evidence of presence of NAbs specific for SARS-CoV-2.

We first compared NAb positivity in relation to disease severity. At the time of first sampling, comparable proportions of asymptomatic individuals (77.8%) and mild COVID-19 patients (69.6%) were circulating neutralizing antibodies suggestive of adequate humoral response of the host. Higher antibody positivity (96.1%, 25 of 26, *P* = 0.03) in the severe COVID-19 patients was noteworthy. Comparison of NAb titers in these groups revealed that the patients with severe disease developed higher titers than in patients with mild disease or asymptomatic infections (Figure 1A). Further, these differences in NAb titers were significant during the first 2 weeks POD (Figure 1D). Clearly, patients with severe disease mounted a higher neutralizing antibody response early in the course of infection. Subsequent comparison was not possible because of the small numbers. We observed similar differences when patients with fulminant hepatitis E were compared with those with mild forms.^20^^,^^21^

Gender-wise comparisons yielded some interesting findings. Symptomatic and severe infections were prominent in males (*P* < 0.001), whereas asymptomatic infection was dominant in females (*P* = 0.0065). Of note, among males, severe-disease patients exhibited higher NAb titers than those with mild disease. The basis for the observed higher symptomatic and severe disease along with higher NAb titers in the males needs to be understood.

Neutralizing antibodies among COVID-19 patients have been measured employing classical PRNT or alternate tests requiring BSL-2-level handling. Among mild-recovered Chinese patients, NAbs were detected from day 10 to 15 POD and ∼30% patients exhibited low levels of neutralizing antibodies.^22^ In a study from the United States (Atlanta, GA), 40 of 44 patients showed neutralization capacity with titers ranging from 1:5,763 to 1:55.^23^ No further details on patients category and/or POD was provided. In a study from New York, NAb titers in hospitalized patients (*n* = 11) were higher than those in outpatients (*n* = 138), with 33% of individuals lacking these neutralizing antibodies; the exact duration of sample collection was not provided.^24^ Overall, lower antibody response was seen in the majority of the COVID-19 patients.

Recent studies have documented higher NAb titers in severe COVID-19 patients.^25^^,^^26^ Importantly, Nab-negative patients (two in the Netherlands and one in our study) remained viral RNA–positive for longer durations. At > 22 POD, decline in antibody titers was found to be rapid in patients with mild disease (Figure 1D). A similar observation was reported by Wang et al. from New York in 35 COVID-19 patients 1 month after symptom onset.^27^ When ELISA was used as a screening test, optical density values obtained for severe disease patients were higher for anti-RBD-IgG (Hong Kong)^11^ and anti-S-IgG (China).^12^ Overall, higher antibodies seem to be associated with disease severity. In this regard, an observation by Quinti et al.^28^ where antibody deficiency was correlated with disease severity is noteworthy. Of seven patients with primary antibody deficiencies and COVID-19 infection, the disease was mild in two patients with agammaglobulinemia (lack of B lymphocytes), whereas the remaining five with common variable immune deficiencies (dysfunctional B lymphocytes) developed severe disease.

We could obtain follow-up blood samples from 18 pateints (including six with fatal outcome) with severe disease (Figure 2B). Unfortunately, serial samples at similar intervals and beyond 3 weeks could not be collected from all the patients. This is a limitation of our study. Nonetheless, irrespective of fatal outcome, most patients with severe disease and longer follow-up exhibited a decline in antibody titers. If sampling is done at this time, an earlier higher rise is likely to be missed. NAb titers did not differ in patients with fatal outcome or recovery. Limited follow-up data for patients with mild disease suggest that the rise in antibody titers was generally low (Figure 2A).

In conclusion, the observed association of NAb titer levels with severity brings up an important issue of whether these antibodies are involved in immunopathology or simply reflect a different cause or marker that is currently unknown. Although correlates of protection against COVID-19 are not yet available, success of plasma therapy in humans^29^ and in animal models^30^ suggest a definite role of antibodies in protection. Data on disease-specific cellular immunity in general and, more specifically, related to disease severity are lacking. In-depth studies relating both arms of immunity in different disease formats are urgently required, especially in view of the expected global immunization efforts as vaccines become available.
